# Newborn screening for acid sphingomyelinase deficiency in Illinois: A single center's experience

**DOI:** 10.1002/jimd.12780

**Published:** 2024-07-11

**Authors:** Rachel E. Hickey, Joshua Baker

**Affiliations:** ^1^ Ann & Robert H. Lurie Children's Hospital of Chicago Chicago Illinois USA; ^2^ Northwestern University Feinberg School of Medicine Chicago Illinois USA

**Keywords:** acid sphingomyelinase deficiency, lyso‐sphingomyelin, newborn screening

## Abstract

Acid sphingomyelinase deficiency (ASMD) is a rare lysosomal storage disorder (LSD) caused by reduced activity of the acid sphingomyelinase (ASM) enzyme, which leads to progressive storage of sphingomyelin and related lipids in the body. ASMD is caused by biallelic variants in the *SMPD1* gene, which encodes for the ASM enzyme. Current estimates of disease incidence range from 0.4 to 0.6 in 100 000 livebirths, although this is likely an underestimation of the true frequency of the disorder. While there is no cure for ASMD, comprehensive care guidelines and enzyme replacement therapy are available, making an early diagnosis crucial. Newborn screening (NBS) for ASMD is possible through measurement of ASM activity in dried blood spots and offers the opportunity for early diagnosis. In 2015, Illinois (IL) became the first to initiate statewide implementation of NBS for ASMD. This study describes the outcomes of screen‐positive patients referred to Ann & Robert H. Lurie Children's Hospital (Lurie). Ten infants were referred for diagnostic evaluation at Lurie, and all 10 infants were classified as confirmed ASMD or at risk for ASMD through a combination of molecular and biochemical testing. Disease incidence was calculated using data from this statewide implementation program and was ~0.79 in 100 000 livebirths. This study demonstrates successful implementation of NBS for ASMD in IL, with high screen specificity and a notable absence of false positive screens.

## 1 INTRODUCTION

Acid sphingomyelinase deficiency (ASMD; also known as Niemann–Pick Disease Type A, Type B or Type A/B; OMIM #257200, #607616) is a rare lysosomal storage disorder (LSD) caused by reduced activity of the acid sphingomyelinase enzyme (ASM, EC 3.1.4.12). This deficiency leads to progressive storage of sphingomyelin and related lipids in the lysosomes of various tissues in the body.^1^ There are 3 subtypes of ASMD, which are differentiated based on age of onset and clinical manifestations: infantile neurovisceral (Type A), chronic visceral (Type B), and chronic neurovisceral (Type A/B). Individuals with Type A disease follow a uniform natural history characterized by severely progressive neurodegeneration, hepatosplenomegaly, failure to thrive, respiratory failure, and death around age 3. Type B disease is associated with a wide clinical spectrum of severity and onset, including hepatosplenomegaly, interstitial lung disease, thrombocytopenia, poor growth, and hyperlipidemia with cardiac disease. Individuals with Type A/B disease present similarly to Type B disease, but will have some degree of neurologic involvement.^2^, ^3^

A diagnosis of ASMD is established by detection of biallelic disease‐causing variants in the sphingomyelin phosphodiesterase 1 (*SMPD1*, OMIM #607608) gene and measurement of deficient ASM enzyme activity. The *SMPD1* gene codes for the ASM enzyme. As of June 2024, 415 variants in the *SMPD1* gene have been reported.^4^ However, in consideration of the known mutation spectrum, there are very few genotype–phenotype correlations available.^5^, ^6^ This is particularly relevant in prognostication and genetic counseling for pre‐symptomatic individuals identified through newborn screening (NBS) programs. Lyso‐sphingomyelin (LSM, also known as Lyso‐SM), a biomarker for ASMD, has been used for monitoring disease progression and treatment efficacy.^7^ Breilyn et al. (2021)^8^ demonstrated a positive relationship between LSM and clinical severity: individuals with Type A disease had higher levels of LSM than those with Type B and Type A/B disease. The use of LSM in prospective phenotyping of pre‐symptomatic individuals has not yet been explored.

While there is no cure for ASMD, there are comprehensive management guidelines to aid in the care of affected individuals^9^ and a newly FDA‐approved enzyme replacement therapy (ERT), olipudase alfa, targeted to the non‐neuronopathic symptoms of the disorder.^10^ Therefore, early diagnosis is crucial to maximizing patient outcomes. Current estimates of disease incidence range from 0.4 to 0.6 cases per 100 000 livebirths, although this is likely an underestimation of the true frequency of the disorder.^11^

NBS for ASMD is possible through measurement of ASM activity in dried blood spots (DBS) and offers the ideal opportunity for early diagnosis of at‐risk individuals. Following a pilot study, Illinois (IL) became the first to implement statewide NBS for ASMD. The first 15‐month experience of NBS for ASMD in IL was previously reported.^12^ This article will extend the initial report and describe the outcomes of ASMD NBS through July 2023.

## 2 MATERIALS AND METHODS

The newborn screening for ASMD in IL was completed in two parts. First, a pilot study screened infants from select birth hospitals within the state. During the pilot phase, DBSs collected on filter card were analyzed dually in the NBS laboratory of the IL Department of Public Health (IDPH) and in the PerkinElmer (Watham, Massachusetts). Results from the PerkinElmer were reported to the referring providers. Later, program expansion to statewide implementation of NBS for ASMD was implemented through measurement of ASM activity via tandem mass spectrometry at the IDPH laboratory.^13^ A positive screen was defined as ASM activity less than 15% of the daily median. In both programs, immediate referral to a designated consultant for diagnostic testing was recommended for infants with a positive screen. Borderline samples between 15% and 20% were asked for repeat sample per protocol. Two borderlines would warrant referral to consultant. This dataset includes all screen‐positive patients referred to Ann & Robert H. Lurie Children's Hospital of Chicago (Lurie) for diagnostic testing, including the pilot program (11/01/2014–05/31/2015) and statewide implementation (06/01/2015–07/31/2023), and accounts for all infants with a positive newborn screen for ASMD in IL. Data collected include results of diagnostic enzyme assay, genotype, confirmed or predicted phenotype, and biomarker values. This retrospective chart review was performed under IRB# 2023‐5864. Additionally, IDPH provided the aggregate number of infants screened during the aforementioned time frames to enable calculation of disease incidence.

**FIGURE 1:**
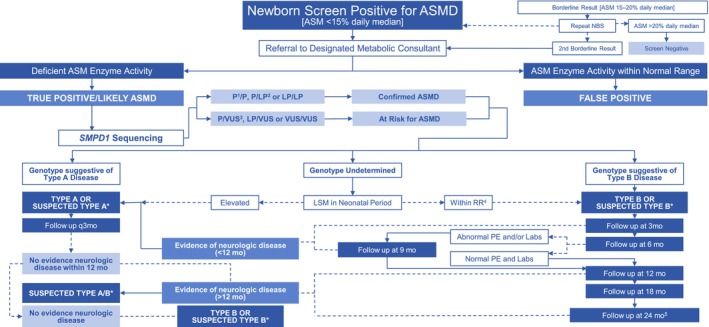
NBS algorithm.

A basic algorithm for diagnosis and follow‐up was created for evaluation of this patient population (Figure 1), adapted from Geberhiwot et al.^9^

## 3 RESULTS

### 3.1 Program totals

A total of 93 792 infants from select birth hospitals in IL were screened during the pilot study for LSD NBS (11/01/2014–05/31/2015). One infant had a positive screen for ASMD: ASM enzyme was measured at 0.75 uM/hr at PerkinElmer [reference range: <0.75 uM/hr]. Statewide implementation of ASMD NBS was initiated on 06/01/2015. A total of 1 137 108 infants were screened between 06/01/2015 and 07/31/2023. Nine infants screened positive for ASMD, with ASM activity measured between 4% and 14% of the daily median. There were no borderline results during the study time frame. In total, 10 infants were referred for diagnostic evaluation at Lurie and all 10 infants were classified as confirmed ASMD or at risk for ASMD through a combination of molecular and biochemical testing (Table 1). The *SMPD1* variants were classified based on the American College of Medical Genetics and Genomics (ACMG) criteria for variant interpretation.^13^ Six infants had confirmed diagnosis due to identification of biallelic pathogenic or likely pathogenic variants. The other four infants are considered at risk for ASMD, due to either the presence of variants of uncertain significance (VUS) or incomplete variant phasing. Disease incidence was calculated using data from the statewide implementation program and was ~1 in 126 345 or ~0.79 in 100 000 livebirths. This incidence was calculated with the expectation that all patients in this cohort will eventually develop disease.

**TABLE 1:** Cohort data.

Demographics of patient population^a^						
	Current age	Self‐reported ancestry	ASM% on NBS^b^	ASM enzyme activity^c^	Genotype^d^	Predicted phenotype^e^
1	9y 10mo	White	PILOT^f^	0.17	Het c.880C > A (p.Q294K) [Path] Het c. 1829_1831delGCC (p.R610del) [Path]	Type B
2	8y 6mo	White	14%	0.24	Het c.880C > A (pQ294K) [Path] Het c. 1829_1831delGCC (p.R610del) [Path]	Type B
3	5y 6mo	Black	6%	0.12	Het c.757G > C (p.D253H) [Path] Het c.689G > A (p.R230H) [VUS]	Type B
4	4y 10mo	White	11%	0.16	Het c.1675_1676del (p.V559Ifs*19) [Path] Het c.872G > A (p.R291H) [VUS]	Type B
5	22 mo	White/Asian	4%	0.24	Het c.175del (p.A59Qfs*18) [Path] Het c.995C > G (p.P332R) [Path]	Type B
6	14 mo	Hispanic	9%	0.45^g^	Het c.1343A > G (p.Y448C) [Path] Het c.1076C > T (p.A359V) [Likely Path]	Type B
7	12 mo	White	10%	0.26	Het c.475 T > C (p.C159R) [Path] Het c.533 T > A (p.I178N) [Likely Path]	Type B
8	10 mo	Asian	5%	0.20	Hom c.995C > G (p.P332R) [Path]	Type B
9	7 mo	White	7%	0.15	Het c.581del (p.P194Qfs*63) [Path] Het c.872G > A (p.R291H) [VUS]	Yet to be determined
10	6 mo	Hispanic/Black	11%	0.40^g^	Het c.1426C > T (p.R476W) [Path] Het c.1132C > T (p.R378C) [Likely Path]	Yet to be determined

^a^ Sex was not included to maintain confidentiality of the cohort.

^b^ Reference Range: >15% uM/L of Daily Median.

^c^ Reference Range: >0.30 nmol/hr/mg protein.

^d^ Reference sequence NM_00543.5.

^e^ Unable to fully differentiate Type B and Type A/B in this young cohort. Long‐term follow up is needed to clarify possible neuropathic involvement.

^f^ Patient 1 screened positive for ASMD through the NBS pilot study, utilizing screening approach.

^g^ Enzyme level was repeated at second laboratory, revealing 0.36 for Patient 6 and 0.18 for Patient 10 (reference range: ≥0.32 nmol/h/mg protein).

### 3.2 Demographics

Basic demographics, enzyme level measured in leukocytes, genotypes, predicted phenotypes, and status (confirmed or at risk for ASMD) are included in Table 1. Individual sex was not included to maintain the confidentiality of this cohort, but 50% of the patients were male. Patients 1–5 and 7–10 were found to have biallelic *SMPD1* variants, confirmed by parental testing. Patient 6 was found to have two *SMPD1* variants that had been previously reported in the literature: Y448C, classified as pathogenic,^14^ and A359V, classified as likely pathogenic.^15^ One parent was positive for the Y448C variant and negative for the A359V variant; the other parent declined carrier screening. Patients 1–5 and 7–9 had ASM enzyme activity reported in the affected range (<0.30 nmol/h/mg protein), ranging 0.12–0.26. Patient 6 and 10 had reported enzyme activity above the affected range at the initial laboratory (0.45 and 0.40, respectively). Repeat measurement at a second laboratory revealed deficient ASM activity for Patient 10: 0.18, normal range ≥0.32 nmol/h/mg protein. Patient 6 had ASM enzyme level above the second laboratory's reported affected range, measured at 0.36.

### 3.3 Observed genotypes

Patients 1 and 2 are full siblings, and the other eight patients are unrelated. Of the nine diverse genotypes that were identified in this cohort, only the sibling pair and Patient 8 had genotypes with a clear phenotype correlation: the siblings are heterozygous for p.R610del, which is suggested to be neuroprotective and Patient 8 is homozygous for the P332R variant, which has been consistently associated with mild Type B disease.^16^ The other seven genotype combinations had not been previously reported in the literature and phenotype prognostication based on genotype alone was not possible. Multiple variants had been reported in patients with both Type A and Type B disease: A59Qfs*18, C159R, Q294K, and Y448C.^14^, ^17^, ^18^, ^19^, ^20^, ^21^ Two variants had only been previously reported in patients with Type A disease: P194Qfs*63 and D253H.^18^, ^22^, ^23^ Seven variants had only been previously reported in patients with Type B disease: I178N, R230H, R291H, A359V, R378C, R476W, and V559Ifs*19.^14^, ^15^, ^24^, ^25^, ^26^, ^27^ Two variants were observed in more than one unrelated patient: R291H and P332R. While R291H and R230H are classified as VUSs by the reference laboratory, both variants have been previously reported in at least one symptomatic patient with ASMD.^25^, ^26^

### 3.4 Categorization of subtype

Patients were broadly categorized as having Type A or Type B disease based on genotype, observed phenotype, and/or biomarker level. Broad categorization was considered necessary for the purposes of genetic counseling and to streamline individual management plans. While patients 1, 2, and 8 were categorized as Type B disease based on genotype, all patients regardless of genotype were monitored closely during their first year of life for evidence of neurologic disease. For those with indeterminate genotypes, determination of either Type A or Type B disease was provided at onset of neurologic dysfunction within the first 12 mo, or after 12 mo with no identified neurologic symptoms. In patients categorized with Type B disease, Type A/B disease could not be excluded due to the young age of this cohort. Patients 3, 4, 5, 6, and 7 were categorized as Type B disease after 12 mo with no identified neurologic symptoms. Patients 9 and 10 are too young to make a determination, at age 7 mo and 6 mo, respectively. LSM was utilized for further evaluation of predicted phenotype and, due to increasing availability of LSM screening tests, several laboratories were used during the study time frame. This biomarker was not commercially available at the initial NBS visit for Patients 1–4. Patient 5 had LSM measured at their first NBS visit: LSM was measured within the normal reference range for this first laboratory. Patients 6–10 had LSM measured via oxysterol panel at their first NBS visit: all patients had LSM measured within the normal reference range for the second laboratory.

### 3.5 Clinical outcomes

The length of follow‐up for the patients ranged from 6 mo to 9 y10 mo. Patients were clinically monitored through a combination of physical exam, laboratory investigations, and imaging studies. At this time, Patients 1 and 2 are the only patients that have been recommended for treatment with ERT. Patient 1 was noted to have abnormal lipid panel at age 8 y 0 mo. Physical exam was not suggestive of organomegaly, but abdominal MRI completed at 9 y7 mo revealed hepatomegaly (931 mL, calculated to 1.4× norm for weight), splenomegaly (351 mL, calculated 7.0× norm), and abnormal marrow signal. Patient 1 started ERT at 9 y 9 mo. Patient 2 first presented with splenomegaly at 18 mo. At age 6, they had massive hepatosplenomegaly precipitating severe gastrointestinal pain, thrombocytopenia, and <3rd percentile for height and weight. While ERT was initially delayed due to pending FDA approval and compassionate use options, Patient 2 started ERT at 6 y 8 mo. Patient 3 had recurrent episodes of epistaxis at age 2, but otherwise normal physical exam, and normal growth parameters. CBC obtained at that time revealed normal platelet level: therefore, epistaxis was considered to be from environmental origin as opposed to early evidence of disease. The other seven patients have normal physical exams, normal laboratory values (CBC, CMP, lipid profile, biomarker), and normal growth parameters. No patients have developed evidence of neurologic disease.

### 3.6 Detection of ASMD carriers

During the study time frame, the family of Patient 4 welcomed two full siblings. Genetic testing completed at birth revealed carrier status for both siblings: one is heterozygous for V559Ifs*19 and the other is heterozygous for R291H. Both siblings screened negative for ASMD on their NBS, with ASM measured at 79% and 91.9% of the daily median, respectively.

## 4 DISCUSSION

### 4.1 Specificity of screen

All ten patients that screened positive for ASMD on NBS were diagnosed with confirmed ASMD or at risk for ASMD through a combination of biochemical and molecular testing. Additionally, at least two known carriers of ASMD screened negative during this study time frame, highlighting the specificity of this screening test. Another factor to be considered when assessing the outcomes of this program is the absence of false positive screens. In the context of newborn screening, a false‐positive screen is defined as an initial out‐of‐range screening result that is ultimately determined to not indicate disease based on additional laboratory evaluations.^28^ NBS for other LSDs, such as Pompe disease, typically have a large number of false positive screens due to the presence of pseudodeficiency alleles in the population, resulting in a significant burden on both the NBS laboratory and designated consultants as well as increased parental anxiety in patients that are ultimately cleared from further follow up.^12^ No evidence of pseudodeficiency for the ASM enzyme was identified in this cohort. While sensitivity cannot be calculated, no cases of false‐negative screens have been identified. Results from this study support the inclusion of ASMD in other NBS programs.

### 4.2 Disease incidence

This study represents the first population‐based screening program for ASMD. The disease incidence was ~0.79 in 100 000 livebirths. It is important to note that this incidence was calculated with the expectation that all patients in this cohort will eventually develop symptoms of ASMD. This calculated incidence is greater than the previously estimated incidence range of 0.4–0.6 in 100 000 livebirths.^11^ It is also notable that the majority of patients have predicted Type B disease. These results suggest individuals with ASMD are underdiagnosed, which may be due to underrecognition of this disorder by clinicians or increased prevalence of the attenuated form of this disorder, which aligns with the NBS outcomes observed for other LSDs.

### 4.3 Genotype–phenotype correlations

In the majority of cases, prognostication based on genotype alone was not possible, complicating genetic counseling of new patients and leading to increased parental anxiety during the first year of life. Multiple variants had been reported in patients with Type A or Type B disease or had only been reported in the compound heterozygous state in one patient, making it difficult to generalize genotype–phenotype correlations. For example, the Y448C variant has been reported in the homozygous state in a patient with Type A disease but also compound heterozygous with R610delin in a patient with Type B disease.^14^ Another variant, V559Ifs*19, has been demonstrated to express a completely inactive truncated protein, which may suggest propensity to severe disease. However, this variant has only been previously reported in the compound heterozygous state with the neuroprotective R610del variant in a patient with Type B disease.^27^ The results of this study emphasize the complexities of genotype interpretation in pre‐symptomatic patients with ASMD and highlight the need for universal registries to facilitate genotype–phenotype predictions.

### 4.4 Use of LSM for categorization of subtype

Six patients had LSM ordered at their initial NBS visit, and all six patients had LSM measured within the laboratory's normal reference range. Patient 8 was predicted to have Type B disease based on genotype. Patients 5–7 did not have any evidence of neurological manifestations within the first 12 mo of life and were categorized to Type B disease using aforementioned broad categorization method, with the caveat that Type A/B disease could not be ruled out due to age. Patients 9 and 10 are <12 mo, but thus far do not have evidence of neurologic disease, such as feeding issues, hypotonia, or failure to thrive. Data from this study may suggest that LSM measured within the normal reference range in the neonatal period is predictive of Type B disease, although it is notable that no patients were categorized as Type A disease in this cohort.

### 4.5 Review of patient 6

Patient 6 presents an interesting case. Genetic testing revealed two heterozygous variants in the *SMPD1* gene, both of which have been previously reported in symptomatic ASMD patients: Y448C, classified as pathogenic,^14^ and A359V, classified as likely pathogenic.^15^ However, this specific genotype combination has not been previously reported in a symptomatic ASMD patient. Only one parent consented to carrier screening for purposes of variant phasing: the Y448C variant was confirmed to be maternally inherited. Although their ASM enzyme level was just above the designated affected range for two separate clinical laboratories, Patient 6 is considered at risk for ASMD. Although their ASM enzyme activity was normal on diagnostic testing, it is notable that the ASM enzyme activity was low enough to have screened positive on the NBS. NBS for LSDs has identified many cases of mild phenotypes that contrast with the classic natural history of a given disorder.^29^ Patient 6's case may be reflective of a mild phenotype, although it will likely be many years before this hypothesis can be confirmed. Nonetheless, this case further emphasizes the specificity of NBS for ASMD: specific enough to identify a patient with a suspicious genotype albeit reported normal ASM enzyme.

### 4.6 NBS for ASMD allows for early and prompt intervention

Previous studies revealed that individuals with ASMD often experience diagnostic delay due to the large phenotypic spectrum of the disorder and overlapping manifestations with other genetic disorders and non‐genetic entities.^2^ Given the availability of management guidelines and therapeutic interventions, early diagnosis of ASMD is a priority for clinicians and families. While clinical trials with olipudase alfa demonstrated significant improvement in visceral disease, affected organ systems did not achieve normal size or functional capacity due to extent of disease burden prior to ERT start.^10^ NBS for ASMD enables early treatment initiation prior to irreversible damage and provides opportunity for increased understanding of the disorder's natural history, leading to improved care and outcomes for affected individuals.

## 5 CONCLUSION

This study demonstrates successful implementation of NBS for ASMD in IL, with high screen specificity and absence of false positive screens. While the lack of genotype/phenotype correlations and prospective biomarker studies pose a significant challenge to prognostication and genetic counseling of pre‐symptomatic newborns, early diagnosis of individuals with ASMD through NBS is anticipated to improve outcomes.

## AUTHOR CONTRIBUTIONS

Joshua Baker contributed to the planning, conduct, and reporting of the work described in the article. Rachel Hickey contributed to the planning, conduct, and reporting of the work described in the article.

## FUNDING INFORMATION

Editorial assistance for this article has been provided by Katy Pocock of GK Pharmacomm Ltd, funded by Sanofi.

## CONFLICT OF INTEREST STATEMENT

Joshua Baker has received speaker honoraria from Sanofi. Rachel Hickey has received speaker honoraria from Sanofi.

## ETHICS STATEMENT

All procedures followed were in accordance with the ethical standards of the responsible committee on human experimentation (institutional and national) and with the Helsinki Declaration of 1975, as revised in 2000 (5). Informed consent was obtained from all patients for being included in the study.

## ANIMAL RIGHTS

This article does not contain any studies with animal subjects performed by the authors.
